# Microfibrillated Cellulose Embedded with KCl as a Solid-Dopant Matrix into an Electrolyte-Gated Transistor

**DOI:** 10.1021/acsomega.5c07536

**Published:** 2026-03-04

**Authors:** Raquel Bettega, Angelo C. Lucizani, Isabela Jasper, Washington L. E. Magalhães, Marcio Vidotti, Keli F. Seidel, José P. M. Serbena

**Affiliations:** † Group of Organic Optoelectronic Devices, Programa de Pós-graduação de Engenharia e Ciência dos Materiais, Universidade Federal do Paraná, Curitiba 81531-980, Brazil; ‡ Grupo de Pesquisa em Macromoléculas e Interfaces, Departamento de Química, Universidade Federal do Paraná, Curitiba 81531-980, Brazil; § Laboratório de Tecnologia da Madeira, Embrapa Florestas, Empresa Brasileira de Pesquisa Agropecuária, Estr. Da Ribeira—Br-476, Colombo, PR 82630-148, Brasil; ∥ Group of Organic Optoelectronic Devices, Departamento Acadêmico de Física, Universidade Tecnológica Federal do Paraná, Curitiba 80230-901, Brasil

## Abstract

Electrolyte retention in electrolyte-gated transistors (EGTs) is typically achieved through viscous electrolytes or extra manufacturing steps for the reservoir design. In this work, we present a multifunctional solid-dopant matrix (SDM) composed of microfibrillated cellulose embedded with potassium chloride (MFC:KCl), which simultaneously acts as an electrolyte reservoir and provides ion anchoring that simplifies the device architecture and processing. For comparison, four electrolyte configurations were systematically investigated: (i) H_2_O (as a nonionic reference), (ii) MFC:H_2_O, (iii) KCl:H_2_O (as an ionic reference), and (iv) MFC:KCl:H_2_O. In water-based transistors, the MFC matrix serves as a pure electrolyte reservoir, showing water retention capability equivalent to the reference device, characterized by an on/off current ratio of ∼10^2^, a threshold voltage of −0.13 V, a maximum drain current of ∼10^–4^ A, and a maximum transconductance of ∼0.5 mS, operating within a stable electrochemical window. In KCl–H_2_O-based transistors, the MFC:KCl material demonstrates dual functionality: simultaneously (i) retaining the electrolyte and (ii) compressing the operational electrochemical window (−0.2 to +0.8 V in MFC:KCl:H_2_O vs −0.9 to +1.0 V in KCl:H_2_O controls). This enables stable transistor operation up to *V*_G_ ∼ −2 V while maintaining comparable current modulation (*I*_on_/*I*_off_ ratios ∼ 10^3^), against unstable operation of KCl:H_2_O electrolyte-based devices. In addition, it presents a threshold voltage of −0.7 V, a maximum drain current of ∼10^–3^ A, and a maximum transconductance of ∼ 3 × 10^2^ mS. This study reveals that MFC offers a versatile platform for both field-effect and electrochemical transistors, aligning with green electronics initiatives by avoiding synthetic polymers like polydimethylsiloxane (PDMS).

## Graphical abstract

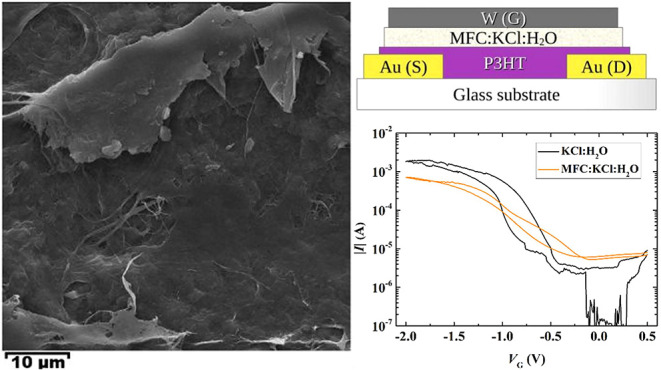

## 1 Introduction

The development and improvement of electrolyte-gated transistors (EGTs) have brought great advances for applications where these devices are applied as biosensors,^1,2^ chemical sensors,^3–5^ neuromorphic devices,^6–8^ and wearable electronics.^9,10^ One of the key enablers of these diverse technologies is the proper coupling and integration of an electrolyte within the transistor architecture. For example, in a sensor, this design allows the analyte to remain in a liquid state, facilitating the presence of mobile anions and cations that can interact with both the organic semiconductor channel and the gate electrode.^11,12^

Among critical parameters to control EGTs’ performance is electrolyte retention on the proper area, which directly reflects the efficiency and reproducibility of the device. To address this challenge, various strategies have been employed, for example, designing and using materials that act as microreservoirs,^13–15^ relying on the intrinsic viscosity of the electrolyte,^8,16^ or using electrolyte matrices that improve the delimitation of the liquid electrolyte area.^17–20^ Considering the last strategy, an abundant and sustainable material that has been successfully applied in electronics is cellulose.^21,22^ In 2008, Fortunato et al.^23^ used cellulose fiber paper as both a substrate and a dielectric in zinc oxide-based field-effect transistors. Other authors used similar approaches using nanocrystalline cellulose as the gate dielectric (NCC) on metal oxide-based transistors,^24^ and cellulose paper with biopolymer chitosan as a substrate,^25^ or chitosan-gated devices in an ethyl cellulose substrate,^26^ or cyanoethyl cellulose^27^ as a substrate, all on organic transistors. In these cases, the devices operated through field effects only, and several strategies were developed to improve the dielectric properties of cellulose, especially considering its surface modification.^28^ To add ionic species to this kind of cellulose-based electrolyte, Thiemann et al.^29^ fabricated microcellulose thin films with tailored 1-ethyl-3-methylimidazolium methylphosphonate ionic liquids, demonstrating working zinc oxide and poly­(3-hexylthiophene-2,5-diyl) (P3HT)-based devices. Cunha et al.^30^ developed cellulose-based composite hydrogel electrolytes (CCHEs) from dissolution of microcrystalline cellulose (MCC) in aqueous lithium hydroxide/urea solvent systems, trapping ions within the cellulose structure and resulting in a cellulose-based hydrogel with high ionic concentrations. Dai et al.^31^ demonstrated the intrinsic ionic conductivity of 2,2,6,6-tetramethylpiperidine-1-oxyl (TEMPO)-oxidized nanocellulose, fabricating solid-state ionic conductive cellulose nanopapers (ICCNs) for use in organic transistors, later developing synaptic^32^ and neuromorphic^33^ devices. More recently, a semiconductor channel based on cellulose and poly­(3,4-ethylenedioxythiophene):poly­(styrenesulfonate) (PEDOT:PSS) was reported by Lee et al.,^34^ demonstrating the versatility of this material.

Recent advancements in electrolyte-gated transistors (EGTs) have prioritized the development of solid-state architectures to overcome the leakage and stability limitations inherent in liquid-gated systems. In this context, the engineering of the electrolyte–semiconductor interface is increasingly reliant on sophisticated polymer matrices and nanocomposites. For instance, blend solid polymer electrolytes (BSPEs) based on poly­(vinyl alcohol) (PVA) and hydroxypropyl methylcellulose (HPMC) doped with CuSO_4_ have demonstrated remarkable ionic conductivity and electrochemical stability.^35,36^ Complementing these synthetic approaches, the synthesis of carboxymethyl cellulose (CMC) from agricultural waste, such as coconut fibers and sugar cane bagasse, offers a sustainable route for producing host polymer membranes with high ionic conductivity (1.37 × 10^–3^ S/cm) and enhanced amorphous regions for efficient ion transport.^37,38^ The mechanical and thermal robustness of such matrices can be further improved through cross-linking strategies involving pyromellitic dianhydride (PMDA) or citric acid, or by the incorporation of aluminum nanorod reinforcements.^38,39^ Moreover, the integration of hybrid nanoparticles, such as polyhedral oligomeric silsesquioxane (POSS), and conducting polymer nanocomposites for selective ion removal further optimizes the structural integrity and functional versatility of these systems.^40,41^ These materials science breakthroughs align with the utilization of microfibrillated cellulose (MFC) as a sustainable solid-dopant matrix, as proposed in this work, providing a multifunctional framework for efficient ion anchoring and electrolyte retention in high-performance EGTs.

Building upon these recent advances in solid-state electrolytes and composite matrices and aiming to contribute to the ongoing efforts in developing sustainable and eco-friendly electronics, this work proposes the integration of a solid-dopant matrix (SDM) composed of microfibrillated cellulose embedded with potassium chloride (MFC:KCl) within the transistor architecture. The novelty lies in the quite simple synthesis and processing of this SDM, along with the use of an industrial consolidated form of cellulose: MFC. The global MFC market size is valued at US$1.28 billion in 2024, with an estimate to be worth US$4.01 billion by 2033.^42^ The proposed approach not only contributes to improving the liquid electrolyte retention but also introduces an additional functional role for the matrix beyond simple containment, which is anchoring salts in its own matrix, allowing aqueous ionic electrolytes.

## 2 Experimental Section

### 2.1 Materials

Interdigitated electrodes composed of Cr/Au (thickness 20 nm/100 nm), patterned onto glass substrates using photolithography, served as the source and drain transistor electrodes. The 50 pairs of digits were spaced by 10 μm and had a width of 3 mm. A tungsten plate, with dimensions of 0.35 × 12 × 25 mm, acted as the gate electrode. The substrates were cleaned in an ultrasonic bath (15 min/each step) in the sequence: acetone, ultrapure deionized water, and isopropyl alcohol. After drying, the substrates were exposed to a UV ozone cleaner for 15 min.

The semiconductor channel consisted of an ∼60 nm poly­(3-hexylthiophene-2,5-diyl) (P3HT) (electronic grade Mw > 45,000 purchased from LUMTEC) thin film, deposited by spin-coating from a 7 mg/mL toluene solution. The thickness was measured with a Bruker XT profilometer. After deposition, the P3HT films were annealed at 100 °C for 60 min in a vacuum oven. Acetone, isopropyl alcohol, toluene, and potassium chloride were purchased from Sigma-Aldrich and used as received.

The MFC membrane is a natural material obtained from organic sources such as wood. It was fabricated via a mechanical disintegration process employing a colloidal mill, a well-established method recognized as one of the most prevalent approaches to produce MFC.^43,44^ The MFC:KCl membrane is a differentiated proposal in this work by the incorporation of KCl during its fabrication stage. This modification facilitates the direct incorporation of the ionic species, commonly employed in the liquid phase diluted in the electrolyte, into the solid-state membrane matrix, thereby enhancing the membrane’s functional performance and enabling the development of the SDM proposed in this study. Cellulose was obtained from bleached eucalyptus pulp, previously fragmented, and dispersed in 3 L of deionized water to form a homogeneous suspension. Potassium chloride was added to this suspension in a 1:1 mass ratio relative to the dry mass of the cellulose. When hydrated, the MFC:KCl:H_2_O electrolyte corresponds to an aqueous KCl solution with a salt concentration of 0.94 mol/L, same concentration used in KCl:H_2_O electrolyte.

### 2.2 Devices Structure and Characterization

The MFC membranes were characterized by using scanning electron microscopy (SEM) and energy-dispersive X-ray spectroscopy (EDS) to assess morphological features and verify the incorporation of potassium chloride into the microfibrillated cellulose matrix. Cyclic voltammetry (CV) studies were conducted on ionic aqueous electrolytes (KCl:H_2_O and MFC:KCl:H_2_O) using an AutoLab PGSTAT204 potentiostat and a regular standard three-electrode cell, where carbon/P3HT comprised the working electrode, and carbon comprised both counter and reference electrodes. The concentration of the KCl:H_2_O electrolyte was 0.07 mg/μL, approximately 7 wt %, while the MFC:KCl membrane was moistened with deionized water, resulting in the electrolyte MFC:KCl:H_2_O with approximately 6.54 wt %. Electrochemical impedance spectroscopy (EIS) measurements were performed in the frequency range from 10,000 to 0.1 Hz, with a perturbation amplitude of 10 mV. The experimental data were analyzed using ZView software, employing equivalent circuit methodology. Transistors’ electrical characterizations were performed on a Keithley 2602 dual source meter unit.

To validate the use of this SDM as a new component in EGTs, four transistors were characterized and compared; their structures are shown in Figure 1. Transistors using deionized water (H_2_O) as the electrolyte retained in the polydimethylsiloxane (PDMS) reservoir were compared with those using microfibrillated cellulose hydrated with deionized water (MFC:H_2_O), aiming to evaluate whether MFC affects transistor performance. On the other hand, transistors with KCl dissolved in deionized water (KCl:H_2_O) retained in PDMS were compared with those using the MFC:KCl functional matrix hydrated with deionized water (MFC:KCl:H_2_O) retained by the SDM itself, to determine if they exhibit similar behavior in standard transistor curves.

**Figure 1:**
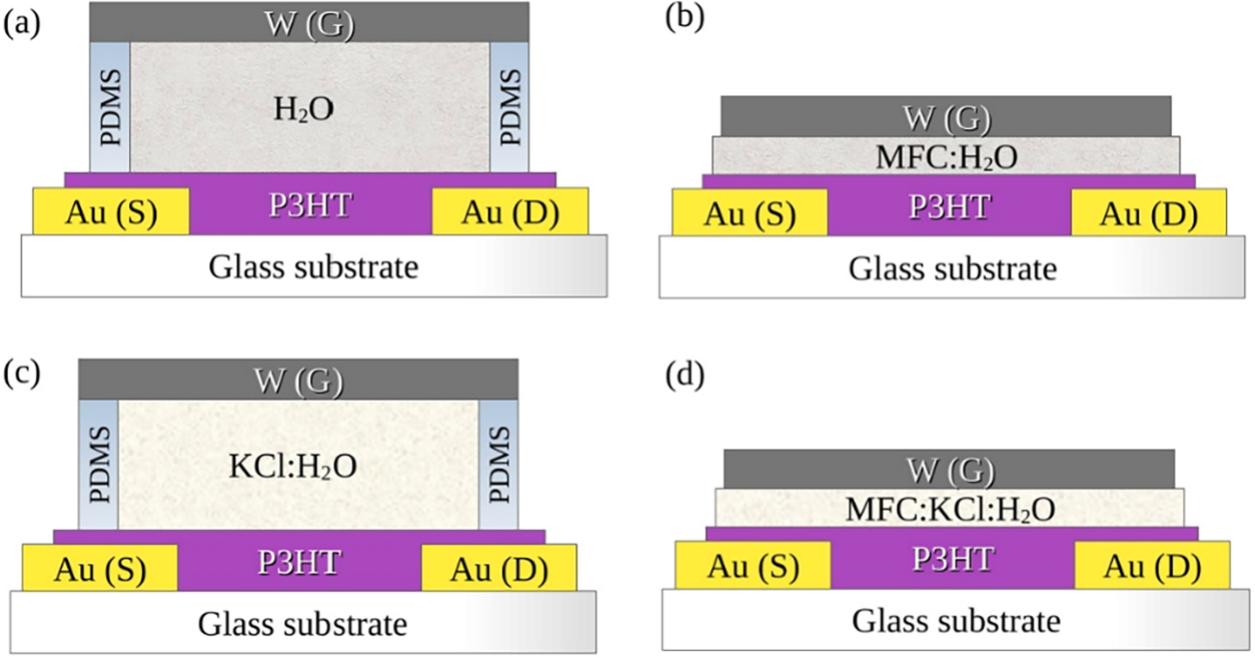
Studied transistor structures, with Au as the source (S) and drain (D) interdigitated electrodes, W as the gate (G) electrode, P3HT as the organic semiconductor channel, and the four compared electrolytes: (a) H_2_O, (b) MFC:H_2_O, (c) KCl:H_2_O, and (d) MFC:KCl:H_2_O (SDM).

The EGT structures are, therefore, planar Au/P3HT/aqueous electrolytes/W. The four aqueous electrolytes were compared: two without ionic species: H_2_O and MFC:H_2_O; and two with ionic species: KCl:H_2_O and MFC:KCl:H_2_O. For the liquid electrolyte transistors (H_2_O and KCl:H_2_O), the PDMS reservoir dimensions were 1 × 4 × 5 mm, resulting in a 20 μL solution volume, while for the solid matrix electrolytes (MFC:H_2_O and MFC:KCl:H_2_O), the MFC membranes have approximately 38 μm thickness, with dimensions of 10 × 15 mm.

## 3 Results and Discussion

Figure 2 presents SEM and EDS mapping images. For MFC surface membranes, SEM images reveal that both MFC (Figure 2a) and MFC:KCl (Figure 2d) samples exhibit a characteristic morphology composed of entangled microfibrils.^45^ EDS mapping (Figure 2b,e) and spectra (Figure 2c,f) further confirm the successful incorporation of KCl into the MFC matrix through the detection of potassium and chloride elemental signals. The salt distribution is homogeneous throughout the sample. In addition, it is possible to observe that no impurities were detected during this analysis, indicating that no ionic species are expected on electrolytes using MFC, and ionic species on MFC:KCl are related to the KCl salt embedded into the membrane.

**Figure 2:**
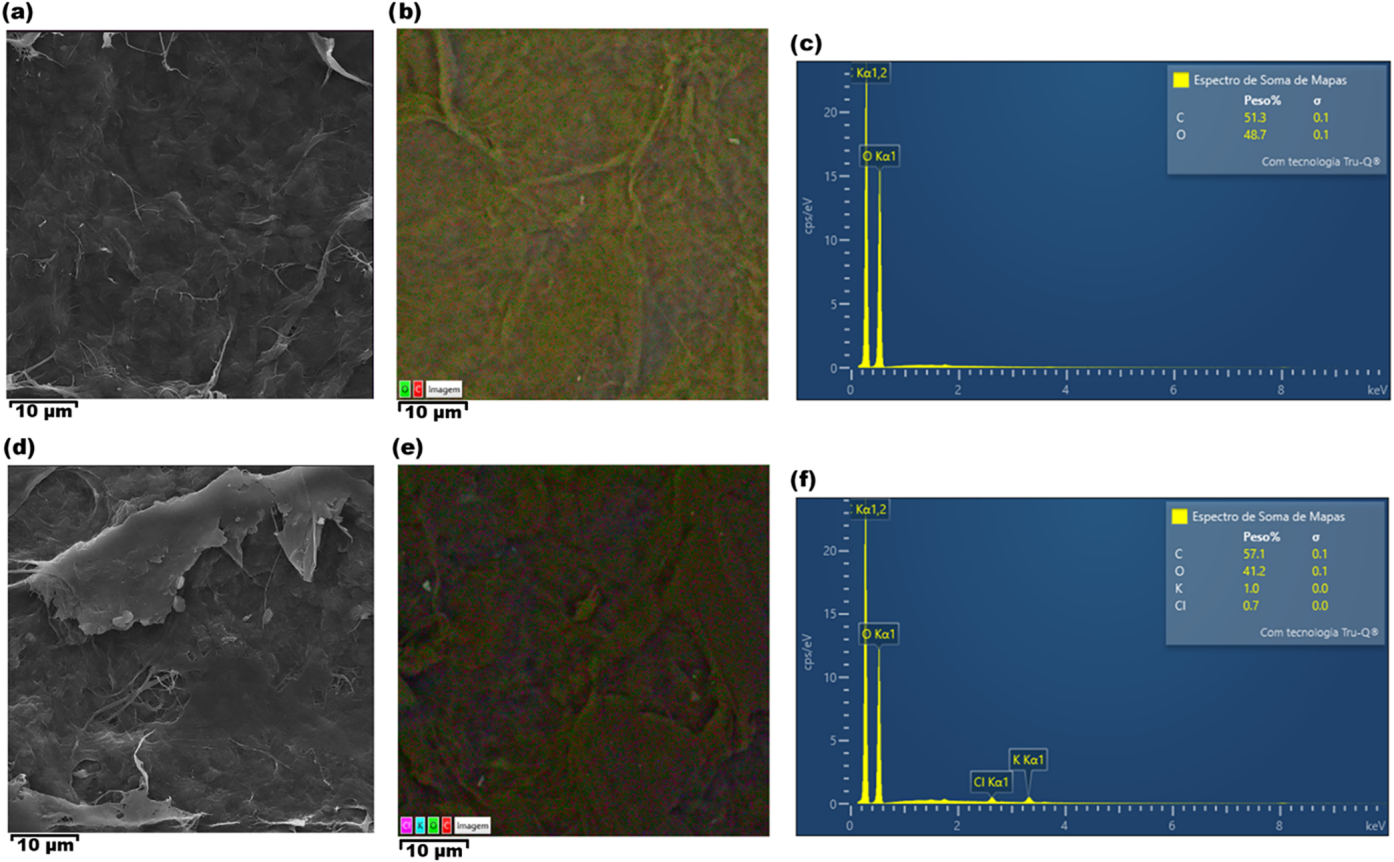
(a) SEM image of pristine MFC; (b) EDS mapping of pristine MFC; (c) EDS spectrum of pristine MFC; (d) SEM image of MFC:KCl; (e) EDS mapping of MFC:KCl; and (f) EDS spectrum of MFC:KCl.

To assess the electrochemical stability of P3HT in different electrolytes, we performed cyclic voltammetry experiments. Figure 3a presents the response of the modified electrode in KCl:H_2_O and MFC:KCl:H_2_O electrolytes at 10 mVs^–1^. Some differences can be pointed out for the MFC:KCl:H_2_O electrolyte: the overall current response is significantly lower and the overall resistive shape of the voltammogram. These aspects can be directly connected with the resistance to ionic transport through the cellulose microfibril (MFC) network, with a decrease in the ionic mobility because of the quasi-solid pathway, in contrast with the many degrees of freedom for a classical aqueous electrolyte. These features are also present when different scan rates are applied to the systems. For KCl:H_2_O, as shown in Figure 3b, the CV profiles exhibit the expected behavior, presenting an increase in the current with the scan rate.^46,47^ On the other hand, the MFC:KCl:H_2_O electrolyte, as shown in Figure 3c, clearly limits the ionic diffusion through in and out of the P3HT solid electrode, maintaining the current almost unaltered even at higher scan rates, prejudicing the ion accessibility and slowing down the charge compensation processes. This regulatory role of MFC may be advantageous in transistor applications, where controlled ion migration can stabilize device operation and minimize unwanted leakage currents.

**Figure 3:**
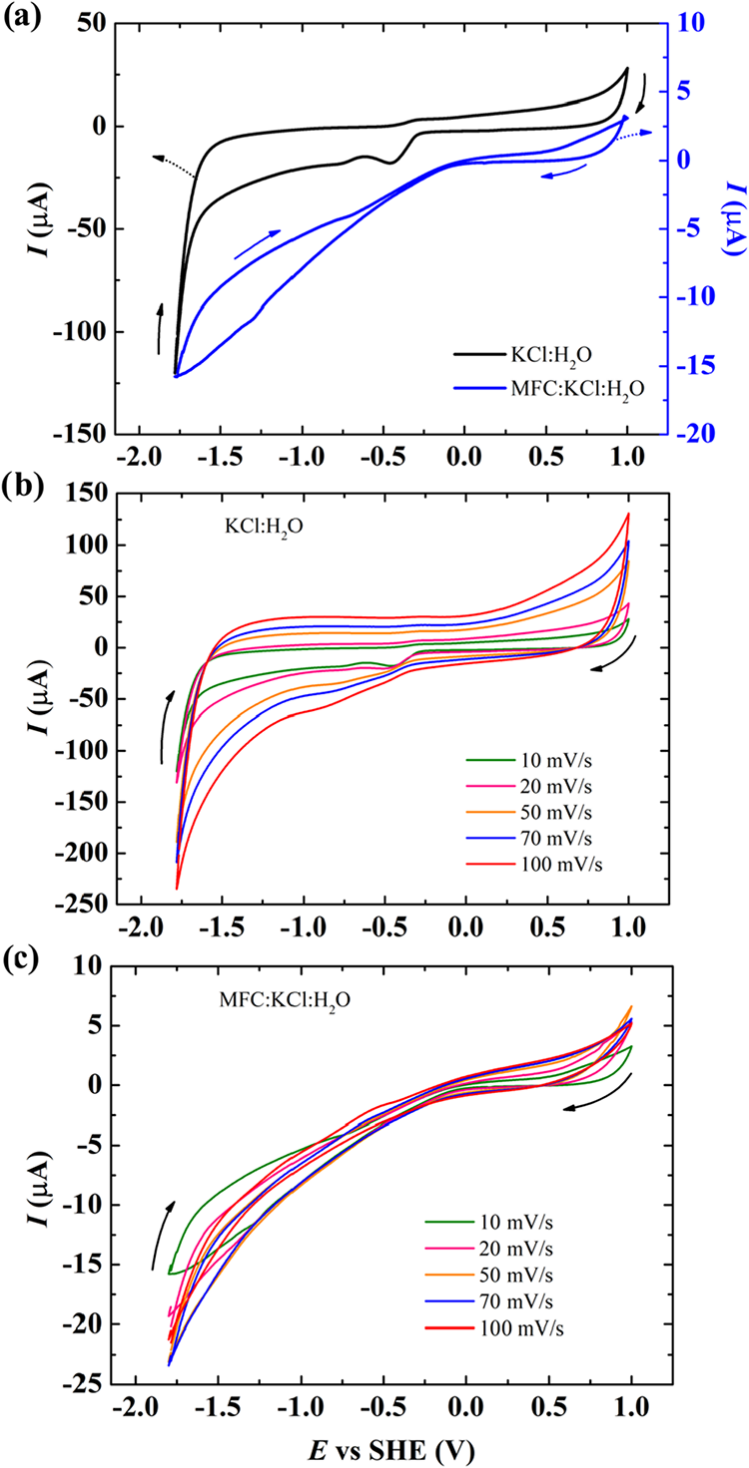
Cyclic voltammetry performed at different scan rates of carbon/P3HT electrodes in different electrolytes. (a) KCl:H_2_O and MFC:KCl:H_2_O at 10 mVs^–1^; (b) KCl:H_2_O electrolyte (10–100 mVs^–1^); and (c) MFC:KCl:H_2_O electrolyte (10–100 mVs^–1^).

To further investigate electrochemical behavior and interfacial effects, electrochemical impedance spectroscopy (EIS) analyses were carried out for both systems, and the Nyquist plots are presented in Figure 4. The experimental data were fitted using the equivalent circuit methodology, as depicted in the inset. It represents a common circuit applied to polymer-modified electrodes,^48,49^ consisting of a series resistance (*R*_s_), a charge transfer resistance (*R*_ct_), and a constant phase element (CPE). *R*_s_ accounts for the sum of the intrinsic resistance of the electroactive material, the electrolyte solution, and the electrical connections; *R*_ct_ is associated with interfacial faradaic processes; and the CPE describes the nonideal capacitive behavior of the electrical double layer.^50–52^

**Figure 4:**
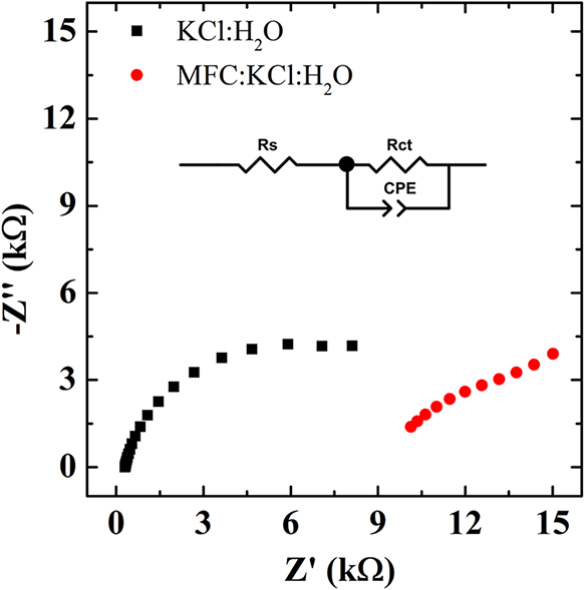
Nyquist plots obtained for the P3HT-modified electrode in different electrolytes.

The fitted parameters are summarized in Table 1. The *R*_s_ values of the MFC:KCl electrolyte are 2 orders of magnitude higher when compared to the KCl:H_2_O system. Since all other experimental conditions (electrolyte composition, electrode spacing, and connections) were the same, the higher *R*_s_ can be directly attributed to the intrinsic electric resistance of the MFC, in agreement with the CV experiments. The charge transfer resistance (*R*_ct_) presented closer values for both electrolytes, indicating that the P3HT interface is potentially suitable for redox reactions, despite the electrolyte studied.

**Table 1:** Equivalent Circuit Fitting Parameters (*R*_s_, *R*_ct_, CPE-T, and CPE-P) Obtained from EIS Measurements of P3HT-Modified Electrodes in Different Electrolytes

**electrolyte**	***R*_s_** (**kΩ**)	**CPE-T** (**F·s** ^***n–*1**^ **)**	**CPE-P**	***R*_ct_** (**kΩ**)
KCl:H_2_O	0.303	3.45 × 10^–5^	0.85	11.9
MFC:KCl:H_2_O	10.33	0.01 × 10^–5^	0.83	8.8

The CPE element, which accounts for the nonideal capacitive behavior of the double layer, showed higher CPE-T values in the systems without MFC, indicating a greater interfacial charge storage capacity, probably due to the higher mobility of the ions on the P3HT interface in aqueous electrolyte, compared to the MFC layer. The CPE-P parameter (ranging from 0 to 1, with values closer to 1 representing an ideal capacitor)^53^ remained similar for both systems, suggesting that the incorporation of MFC did not significantly alter the overall homogeneity of the electrode/electrolyte interface and that the charge distribution process remained largely uniform regardless of the electrolyte composition. Overall, the incorporation of MFC significantly increases the resistance to both electronic and ionic transport^54–56^ while decreasing the effective capacitance of the double layer.^57,58^ These findings highlight the regulatory role of the MFC network in modulating ion transport and mitigating uncontrolled charge dynamics, in agreement with the cyclic voltammetry results.^59,60^

Figure 5a shows the typical transfer curves (|*I*_DS_| vs *V*_G_) of water-based EGTs with H_2_O (red line symbol) and MFC:H_2_O (blue line symbol) electrolytes at a scan rate of 100 mV/s. The H_2_O-only device serves as a reference, highlighting the role of the MFC in the electrolyte. The nearly identical performance of the two devices suggests that MFC acts primarily as a passive electrolyte-retention matrix, with no significant impact on charge transport. However, minor hysteresis is observed in the MFC:H_2_O device. Since EDS analysis (Figure 2b,c) rules out ionic impurities, it is plausible to attribute this hysteresis to delayed interfacial polarization, where MFC’s hydroxyl-rich surface and bound water molecules hinder dipole reorientation at the semiconductor/electrolyte interface under gate bias. The dielectric behavior of hydrated cellulose stems from its polysaccharide structure, where hydroxyl groups (C2, C3, and C6) create localized dipoles that interact strongly with water molecules through hydrogen bonding. These interactions restrict molecular mobility while enhancing interfacial polarity, forming a dynamically correlated network where water dipoles preferentially orient toward hydrophilic troughs between cellulose chains, whereas hydrophobic C–H regions induce water self-clustering. The conformation of hydroxymethyl groups governs this interfacial water structuring, which in turn modulates wetting properties and creates hydration-dependent electrostatic fields.^61^ This bidirectional coupling, where cellulose’s hydroxyl groups direct water dipole alignment through electrostatics, while the oriented water molecules stabilize cellulose surface dynamics via hydrogen-bond networks, underpins the unique dielectric and hydration characteristics essential for electrolyte-gated transistor applications. Importantly, this mechanism enables MFC to replace conventional materials like PDMS while offering additional advantages such as simplified processing and inherent environmental sustainability.

**Figure 5:**
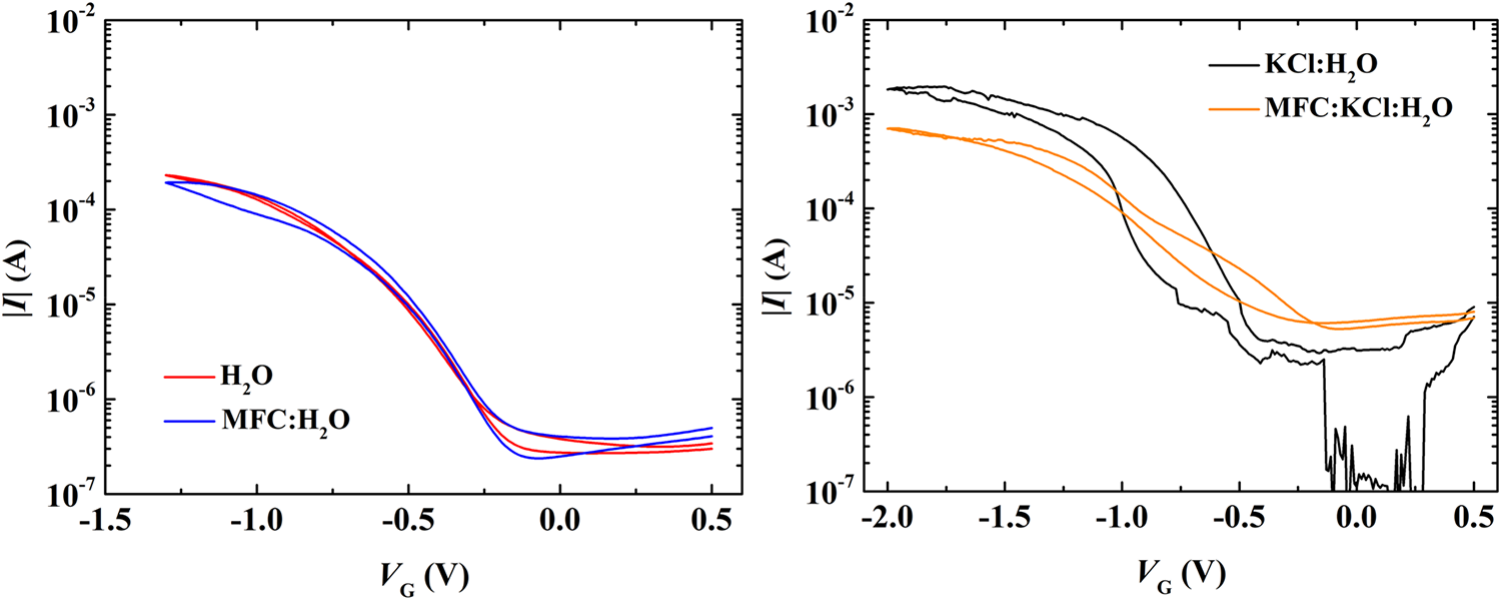
Transfer curves of the studied devices at *V*_DS_ = −0.8 V. Electrolytes (a) absent on ionic species: H_2_O (red) and MFC:H_2_O (blue) at a scan rate of 100 mV/s; and (b) present on ionic species: KCl:H_2_O (black) and MFC:KCl:H_2_O (orange) at a scan rate of 10 mV/s.

Figure 5b shows the transfer curves of EGTs with KCl:H_2_O (black line symbol) and MFC:KCl:H_2_O (orange line symbol) electrolytes, with a scan rate of 10 mV/s. The KCl:H_2_O device serves as a reference to isolate the role of the MFC in the ionic electrolyte. In the MFC:KCl:H_2_O system, KCl dissociation occurs only upon hydration in situ, whereas the KCl:H_2_O electrolyte is predissociated. This difference suggests that ion dynamics (e.g., dissociation kinetics and transport through MFC’s nanoporous matrix) may govern device time scales and stability. A slower scan rate was employed to ensure steady-state ion-channel interactions, and an extended negative *V*_G_ range was used to resolve the saturation behavior. Despite a modest current reduction, the MFC:KCl:H_2_O device exhibited superior operational stability and reduced hysteresis compared to the KCl:H_2_O device. These observations imply that MFC (i) partially restricts ion release upon hydration (evidenced by lower *I*_on_) and (ii) modulates ion transport due to the decreased channel area for ionic doping. The instability of the KCl:H_2_O device at positive *V*_G_ aligns with reports of irreversible Faradaic processes in unbuffered aqueous EGTs,^62^ while MFC’s porosity may mitigate such effects by limiting the liquid phase flux (predominantly solubilized ion flux).^61^ These results highlight MFC’s dual role as an ion modulator and an interfacial stabilizer in EGT’s accumulation mode.

Figure 6 presents typical output curves (*I*_DS_ vs *V*_DS_) of all transistors measured under ambient conditions: nonionic (a) H_2_O and (b) MFC:H_2_O, *V*_G_ ranging from +0.1 to −0.9 V, and ionic (c) KCl:H_2_O and (d) MFC:KCl:H_2_O, *V*_G_ ranging from 0 to −1.75 V. The curves show modulation of channel current dependent on gate voltage, which confirms effective gating through both nonionic and ionic hydrated MFC.

**Figure 6:**
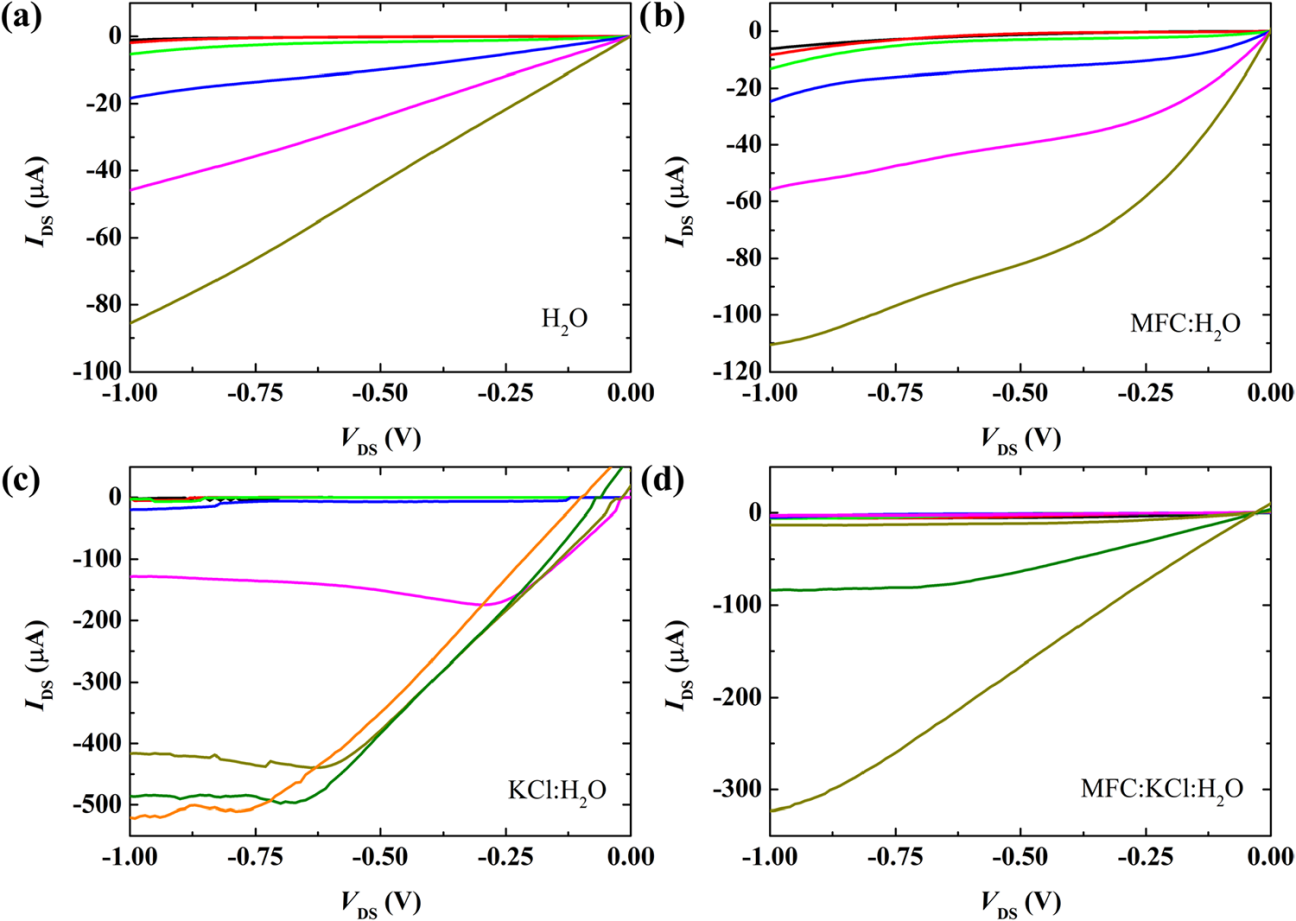
Typical output curves of the fabricated devices comparing the four studied electrolytes: (a) H_2_O; (b) MFC:H_2_O; (c) KCl:H_2_O; and (d) MFC:KCl:H_2_O. Gate voltage (*V*_G_) varies from (a) and (b) 0.1 to −0.9 V and (c) and (d) 0.0 to −1.75 V.

Devices utilizing nonionic electrolytes show similar drain current magnitudes, consistent with their transfer curves, but slightly distinct output characteristics. The H_2_O electrolyte prevents the output curves from reaching saturation at high gate voltages, indicating difficulty in channel pinch-off. Conversely, the MFC:H_2_O system approximates saturation but exhibits significant current drift. The absence of clear saturation current in these systems can be attributed to contact barriers between source/drain electrodes and channel semiconductor.^63,64^ For the ionic systems, KCl:H_2_O devices present higher current magnitudes than MFC:KCl:H_2_O devices, also matching the transfer curve results. Both ionic systems transition into the saturation regime. However, this transition is slightly shifted toward higher *V*_DS_ when the MFC is present. This shift in saturation voltage can be directly correlated with the higher threshold voltage observed for the MFC-containing devices, suggesting an increased trap-filling requirement for channel opening.^65^ In addition, it is possible to observe the instability of the KCl:H_2_O devices at more negative gate voltages.

The electrical parameters extracted from the devices further demonstrate their performance compatibility, as summarized in Table 2. It is important to note that *I*_on_ was extracted at *V* ^_G_^ = −1.3 V for non-ionic electrolytes, while *V*_G_ = −2.0 V was used for ionic electrolytes. Nevertheless, the almost 1 order of magnitude higher achieved values in ionic electrolytes than in nonionic ones may be attributed mainly to the ionic species, present in one type of electrolyte and absent in the other. Similar difference values are observed for the *I*_off_. Therefore, the ionic species increases not only *I*_on_ but also *I*_off_, making all devices present similar *I*_on_/*I*_off_ ratios. Finally, threshold voltages (*V*_T_) presented very similar values, except for KCl:H_2_O-based devices, in which lower values are measured.

**Table 2:** Electrical Parameters of the Studied Devices Extracted from the Transfer Curves

**electrolyte**	***I***_**on**_ **/** ***I***_**off**_	***V***_**T**_ **(V)**	***g***_**m**_ **(mS)**
H_2_O	∼3 × 10^2^	–0.11 ± 0.02	(0.48 ± 0.06)
MFC:H_2_O	∼6 × 10^2^	–0.13 ± 0.02	(0.5 ± 0.2)
KCl:H_2_O	∼5 × 10^3^	–0.80 ± 0.08	(45 ± 5) × 10
MFC:KCl:H_2_O	∼3 × 10^3^	–0.7 ± 0.2	(3 ± 2) × 10^2^

## 4 Conclusions

In this study, we demonstrated that microfibrillated cellulose (MFC) is a highly effective and sustainable material for electrolyte management in aqueous electrolyte-gated transistors. The proposed solid-dopant matrix (SDM), composed of MFC embedded with KCl (MFC:KCl), proved to be multifunctional, acting simultaneously as a stable electrolyte reservoir and providing efficient ion anchoring. Our results showed a clear distinction in performance between the investigated systems. In nonionic electrolytes, the MFC matrix exhibited no additional electrical effect on the transistors, characterized by *I*_on_/*I*_off_ ratios of ∼10^2^, a *V*_T_ of −0.13 V, a maximum *I*_D_ of ∼10^–4^ A, and a *g*_m_ of ∼0.5 mS. In ionic electrolytes, the MFC:KCl-gated EGTs significantly improved device performance, achieving *I*_on_/*I*_off_ ratios of ∼10^3^, a *V*_TH_ of −0.7 V, a maximum *I*_D_ of ∼10^–3^ A, and a *g*_m_ of ∼3 × 10^2^ mS. These findings confirm that the MFC:KCl SDM enables the device to operate within a stable and compressed electrochemical window, simplifying the transistor architecture by eliminating the need for complex electrolyte containment. In conclusion, the integration of this cellulose-based material not only contributes to the development of eco-friendly electronics but also provides a scalable and high-performance pathway for the next generation of sustainable organic devices.

## Data Availability

The data that support the findings of this study are available from the corresponding author upon reasonable request.
